# Implementation Leadership in Evidence‐Based Practice in Healthcare: A Systematic Review and Meta‐Analysis

**DOI:** 10.1111/wvn.70163

**Published:** 2026-08-10

**Authors:** Suvi Kuha, Jouko Miettunen, Outi Kanste

**Affiliations:** ^1^ Research Unit of Health Sciences and Technology, Faculty of Medicine University of Oulu Oulu Finland; ^2^ The Finnish Centre for Evidence‐Based Health Care: A Joanna Briggs Institute Centre of Excellence Helsinki Finland; ^3^ Research Unit of Population Health University of Oulu Oulu Finland; ^4^ MRC Oulu, Oulu University Hospital, University of Oulu Oulu Finland; ^5^ Research Unit of Health Sciences and Technology University of Oulu Oulu Finland

**Keywords:** evidence‐based practice, healthcare, implementation leadership, meta‐analysis, nursing, systematic review

## Abstract

**Background:**

Evidence‐based practice (EBP) is essential in contemporary high‐pressure healthcare environments, yet its implementation remains challenging. Despite the pivotal role of leadership, the lack of pooled quantitative evidence limits understanding of implementation leadership levels and the identification of development needs.

**Aim:**

To investigate the pooled mean levels of implementation leadership in EBP among healthcare professionals and managers.

**Design:**

Systematic review and meta‐analysis.

**Methods:**

This systematic review followed the Joanna Briggs Institute methodology and utilized the PRISMA statement. Findings were synthesized using random‐effects meta‐analyses of mean levels. Heterogeneity was assessed using the *I* ^2^ statistic. A systematic search was conducted in November 2025 in MEDLINE (Ovid), CINAHL, Scopus, Web of Science, ProQuest, and Medic with no date or geographical limits. Studies in English, Finnish, and Swedish were included.

**Results:**

A total of 22 studies published between 2014 and 2025 across nine countries, involving 9970 participants, were included. The overall pooled mean level of implementation leadership in EBP, measured using the Implementation Leadership Scale (range 0–4), was 2.66 (95% CI: 2.49–2.83). Subscale pooled means were highest for supportive leadership (3.00, 95% CI: 2.83–3.16), followed by knowledgeable (2.78, 95% CI: 2.57–3.00), perseverant (2.77, 95% CI: 2.58–2.96), and proactive leadership (2.43, 95% CI: 2.25–2.61). Subgroup analyses showed pooled means of 2.47 (95% CI: 1.98–2.97) for managers and 2.70 (95% CI: 2.52–2.88) for professionals. Considerable heterogeneity was observed across studies.

**Linking Evidence to Action:**

Strengthening implementation leadership to support EBP is needed across healthcare. Organizations should invest in structured, targeted leadership development initiatives that cultivate supportive, knowledgeable, perseverant, and especially proactive leadership behaviors, while ensuring programmes are context‐sensitive and adaptable to variability across healthcare settings. In addition, role‐based differences highlight the need for tailored support for managers that reflect the complexity and accountability of their leadership responsibilities.

**Trial Registration:**

PROSPERO ID: [CRD420251241584]

## 1 Introduction

Healthcare systems are increasingly complex, requiring adaptive practices and strategic resource allocation to ensure high‐quality and efficient care (Rajit et al. 2024), while evidence‐based practice (EBP) remains essential for informed decision‐making, integrating the best available evidence with clinical expertise and patient preferences and values (Jordan et al. 2019; Melnyk and Fineout‐Overholt 2019). Prioritizing EBP enables healthcare organizations to respond to evolving demands while supporting optimal patient outcomes, care quality, and health equity (Connor et al. 2023; Dopp et al. 2024).

Despite its benefits, EBP implementation continues to face persistent challenges (Bauer and Kirchner 2020; Connor et al. 2023), and evidence‐based guidelines alone are insufficient to close the evidence–practice gap. This has increased the emphasis on intentional, theory‐informed implementation strategies (Bauer and Kirchner 2020), as nurses' EBP beliefs and engagement are shaped by individual and organizational factors (Mehra et al. 2025). Implementation in healthcare is commonly conceptualized through frameworks such as the Exploration, Preparation, Implementation, and Sustainment (EPIS) model (Aarons et al. 2011) and the Joanna Briggs Institute Model of Evidence‐Based Healthcare (Jordan et al. 2019), both of which emphasize implementation as an ongoing process shaped by contextual influences and highlight the central role of leadership across implementation phases.

Leadership is widely recognized as a key mechanism for operationalizing and sustaining implementation strategies (Kitson et al. 2021; Nordin et al. 2022; Williams et al. 2020). Implementation leadership (IL) refers to a strategic set of leader behaviors that facilitate the effective implementation of EBP (Castiglione 2020). Given the pivotal role of leadership in facilitating implementation, researchers have emphasized the need for robust, theory‐driven instruments to assess leadership behaviors specifically aligned with EBP implementation (e.g., Aarons et al. 2014; Castiglione 2020).

The most extensively validated instrument for assessing IL is the Implementation Leadership Scale (ILS) (Aarons et al. 2014), comprising four subscales: proactive, knowledgeable, supportive, and perseverant leadership. Proactive leadership involves anticipating and addressing implementation challenges; knowledgeable leadership reflects leaders' understanding of EBP; supportive leadership encompasses behaviors that encourage and enable EBP adoption; and perseverant leadership captures leaders' capacity to sustain implementation efforts despite obstacles.

The psychometric properties of the ILS have been validated in diverse healthcare contexts (Egeland et al. 2023; Finn et al. 2016; Williams et al. 2020) and across different languages and cultural settings (Braathu et al. 2022). Despite this, notable variation in ILS mean scores has been reported. High scores—particularly for supportive leadership—have been observed in U.S. child welfare services (Finn et al. 2016), whereas moderate scores have been found in U.S. child mental health services (Williams et al. 2020) and lower scores in Norwegian mental health settings (Egeland et al. 2023). Furthermore, studies indicate that managers tend to rate their own leadership behaviors more positively than professionals do, underscoring systematic perceptual differences (Torres et al. 2017).

IL is consistently associated with favorable EBP‐related outcomes, including improved attitudes (Sklar et al. 2024), stronger implementation climate and culture (Egeland et al. 2023; Sklar et al. 2024), and enhanced organizational readiness (Egeland et al. 2023). At the same time, deficits in IL are widely documented across healthcare contexts (Cleary‐Holdforth et al. 2021; Gifford et al. 2019), and both managers and professionals frequently report limited confidence in their ability to implement EBP (Koivunen et al. 2024; Nwe et al. 2024). Such deficits contribute to mis‐implementation and hinder the integration of research evidence into routine care (Dopp et al. 2024).

Although IL is recognized as critical to successful EBP implementation, existing reviews have primarily focused on broader implementation determinants rather than leadership itself. Earlier reviews identified leadership as a key organizational determinant of EBP (Li et al. 2018), and more recent reviews show that multiple implementation strategies improve outcomes across healthcare settings (Zhang et al. 2025). Searches of PROSPERO, PubMed, the Cochrane Database of Systematic Reviews, and JBI Evidence Synthesis identified no published or ongoing systematic reviews specifically examining levels of IL in EBP within healthcare. Consequently, evidence remains fragmented; quantitative observational studies are underrepresented, and no meta‐analysis has been conducted, limiting understanding of IL levels and related development needs.

## 2 The Review

### 2.1 Aim

This systematic review aims to investigate the pooled mean levels of implementation leadership in evidence‐based practice among healthcare professionals and managers.

### 2.2 Design

This systematic review followed the Joanna Briggs Institute (JBI) guidelines (Aromatis and Munn 2020) and utilized the PRISMA statement for reporting (Table S1). The review protocol was registered at PROSPERO [CRD420251241584].

### 2.3 Search Methods

A three‐step search strategy was employed. First, an initial limited search of MEDLINE (Ovid) and CINAHL was conducted, followed by an analysis of text words in titles and abstracts along with index terms. The search strategy was adapted for each information source. A second, comprehensive search was conducted in November 2025 across MEDLINE, CINAHL, Scopus, Web of Science, ProQuest, and the Finnish database Medic, with no date limits (Table S2). The strategy was developed in collaboration with an information specialist. Finally, reference lists of the studies were screened for additional relevant studies. Only studies published in English, Finnish, or Swedish were included due to language proficiency and translation constraints.

### 2.4 Inclusion and Exclusion Criteria

Inclusion and exclusion criteria were defined based on the PEO (Participants, Exposure, and Outcome) guidelines (Munn et al. 2018; Table 1).

**TABLE 1:** Inclusion and exclusion criteria (PEO).

PEO	Inclusion criteria	Exclusion criteria
Participants (P)	Professionals (e.g., nurses, physiotherapists, occupational therapists, physicians) and managers, with at least 50% of the sample representing these groups	Healthcare students, patients, and other professional groups not working within healthcare settings Mixed samples including professionals and managers
Exposure of interests (E)	Implementation leadership in evidence‐based practice	Other leadership behaviors or general implementation processes not explicitly linked to evidence‐based practice
Outcome (O)	Pooled mean levels of implementation leadership in evidence‐based practice, including, but not limited to, standardized measures such as the Implementation Leadership Scale (ILS)	Measures focusing on implementation or leadership at a general level
Type of studies	Quantitative cross‐sectional original studies and mixed‐methods studies, if quantitative cross‐sectional data can be extracted from the qualitative components	Qualitative studies, longitudinal study designs, reviews, summaries, documentaries, discussion or opinion papers

*Source:* Authors own work.

### 2.5 Study Selection

**FIGURE 1:**
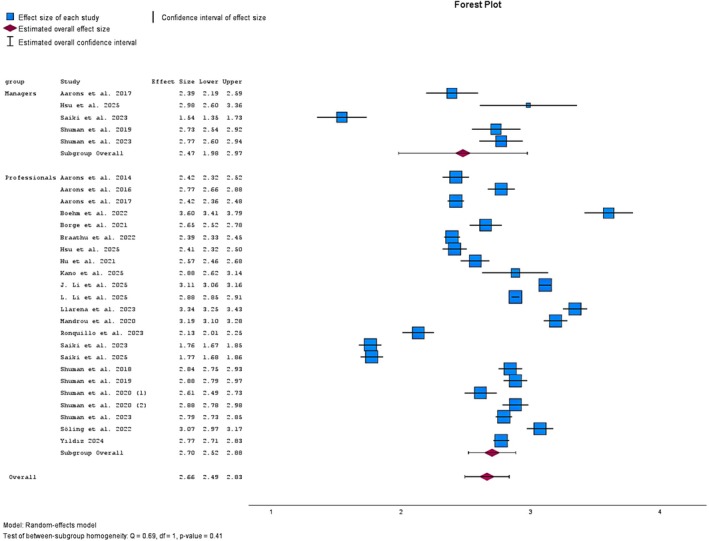
Meta‐analysis of mean levels of implementation leadership in evidence‐based practice in healthcare.

The database search identified 699 studies. All identified citations were collated and uploaded into the Covidence bibliographic management system 2.0, where 444 duplicates were identified and removed. Following a pilot test, two independent reviewers (SK, OK) screened the titles and abstracts based on the inclusion criteria. A total of 68 full texts were assessed for eligibility, and after excluding 46 irrelevant studies (Table S3), 22 studies were included (Figure 1). The reference lists of all included studies were manually reviewed for additional relevant studies; however, no further studies were identified. Any disagreements that arose between the reviewers were resolved through discussion.

### 2.6 Quality Appraisal

Two independent reviewers (SK, OK) evaluated the methodological quality of 22 studies using a standardized critical appraisal checklist from JBI for Analytical Cross‐sectional studies (Moola et al. 2020). Any disagreements that arose between the reviewers were resolved through discussion. All studies, regardless of methodological quality, underwent data extraction and synthesis. The quality assessment scores included in the studies ranged from 6 to 8 out of 8 points, indicating high quality (Table S4).

### 2.7 Data Extraction

Data were extracted from the included studies using a data extraction tool created by the researchers in line with JBI guidelines (Aromatis and Munn 2020). The first author (SK) extracted all relevant data, which was then cross‐checked by another author (OK). The extracted data included detailed information about the authors, publication year, country, participants, data collection, data analysis, measures, and key findings related to the review questions (Table S5).

### 2.8 Statistical Analysis

A random‐effects meta‐analysis was conducted to pool mean estimates of IL using the Implementation Leadership Scale (ILS) (Aarons et al. 2014), as other IL measures were unsuitable for meta‐analysis due to methodological heterogeneity and limited or mixed samples. A random‐effects meta‐analysis (Tufanaru et al. 2015) was performed in IBM SPSS Statistics 29.0, including total ILS scores and the four subscales (proactive, knowledgeable, supportive, and perseverant), both overall and by professional role. Heterogeneity was assessed using Cochran's *Q* and the *I* ^2^ statistic, with *I* ^2^ values above 50% indicating substantial heterogeneity (Higgins and Thompson 2002).

## 3 Results

### 3.1 Characteristics of Included Studies

The studies (*n* = 22) were published between 2014 and 2025 in the United States (*n* = 10), China (*n* = 3), Norway (*n* = 2), Canada (*n* = 1), Germany (*n* = 1), Greece (*n* = 1), Spain (*n* = 1), Türkiye (*n* = 1), and Japan (*n* = 2). The number of participants ranged from 86 to 2124, totaling 9970. Participants were healthcare professionals (e.g., nurses, midwives) and managers (e.g., supervisors, nurse managers) across various management levels. Most of the studies were conducted in hospital settings (Table S5).

### 3.2 Overall Pooled Levels of Implementation Leadership in Healthcare

**FIGURE 2:**
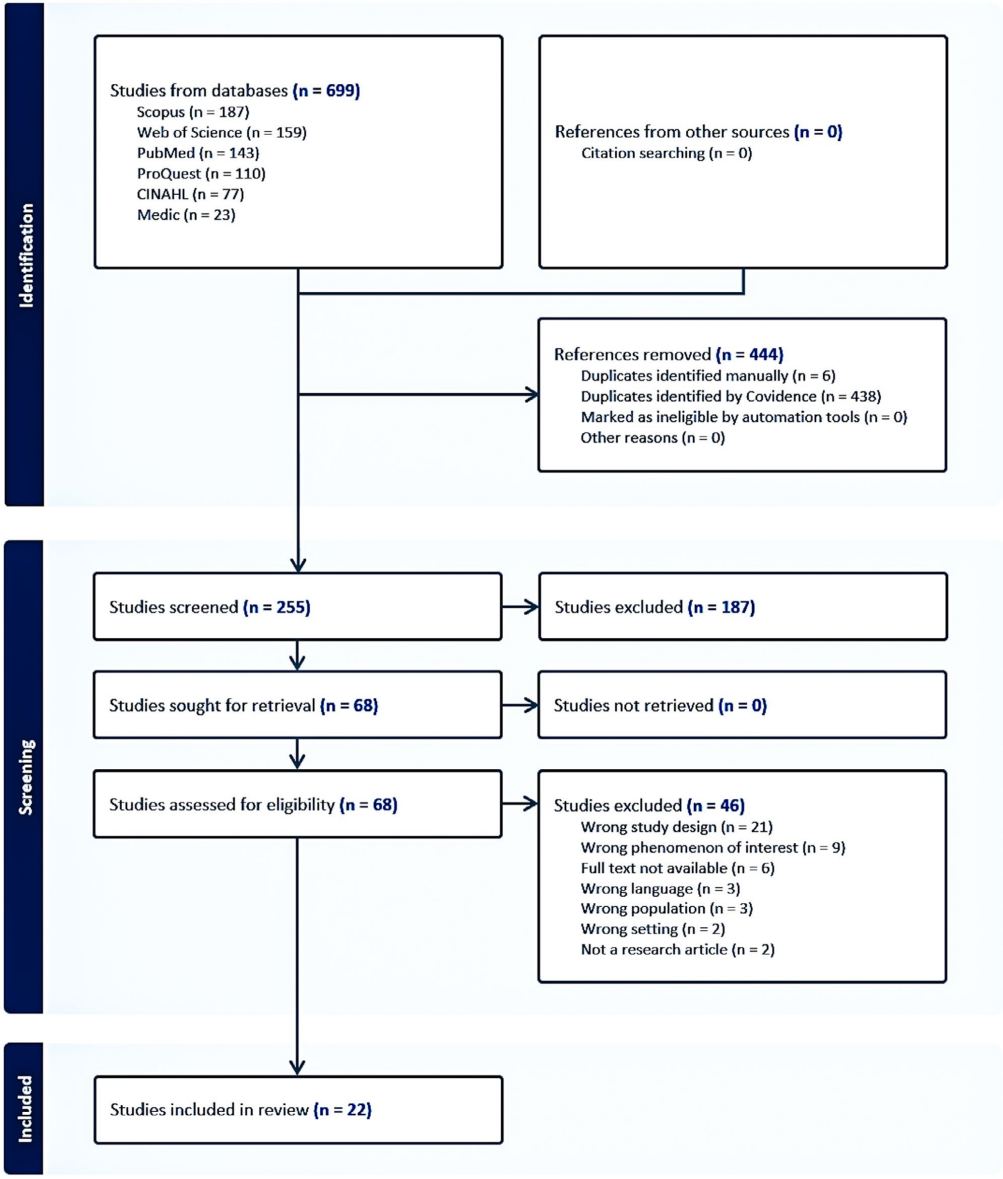
PRISMA flowchart of the search results, study selection, and inclusion process.

In the meta‐analysis, the overall pooled mean level of IL, measured using the Implementation Leadership Scale (ILS; range 0–4), was 2.66 (95% CI [2.49–2.83], *z* = 30.30, *p* < 0.001) (Figure 2). Substantial heterogeneity was observed across the included studies. The overall Cochran's *Q*‐test indicated statistically significant heterogeneity (*Q* = 2236.86, df = 27, *p* < 0.001), accompanied by a high *I* ^2^ value (*I* ^2^ = 99.1%).

Subscale analyses indicated that the highest pooled mean level was observed for supportive leadership (mean = 3.00, 95% CI [2.83–3.16], *z* = 35.77, *p* < 0.001), followed by knowledgeable (mean = 2.78, 95% CI [2.57–3.00], *z* = 25.12, *p* < 0.001), perseverant (mean = 2.77, 95% CI [2.58–2.96], *z* = 29.06, *p* < 0.001), and proactive leadership (mean = 2.43, 95% CI [2.25–2.61], *z* = 26.61, *p* < 0.001) (Table 2). Heterogeneity was consistently high across all subscales (*Q* = 933.48–1583.05; *I* ^2^ > 90%, *p* < 0.001).

**TABLE 2:** Pooled means of the subscales of implementation leadership.

Subscale	Subgroup		Overall pooled mean (95% CI) (22 studies)
	Managers pooled mean (95% CI) (5 studies)	Professionals pooled mean (95% CI) (22 studies)	
Proactive	2.08 (1.57–2.60)	2.50 (2.32–2.68)	2.43 (2.25–2.61)
Knowledgeable	2.38 (1.81–2.96)	2.87 (2.64–3.09)	2.78 (2.57–3.00)
Supportive	3.07 (2.44–3.69)	2.98 (2.82–3.15)	3.00 (2.83–3.16)
Perseverant	2.57 (1.92–3.22)	2.81 (2.62–3.00)	2.77 (2.58–2.96)

Abbreviation: CI, confidence interval (lower, upper).

*Source:* Authors own work.

Means for individual studies, including subscale estimates with 95% confidence intervals, are provided in the Supporting Information (Tables S6–S10).

### 3.3 Pooled Implementation Leadership Levels for Professionals and Managers

The subgroup‐specific pooled mean levels of IL were 2.47 (95% CI [1.98–2.97], *z* = 9.75, *p* < 0.001) in the manager's subgroup and 2.70 (95% CI [2.52–2.88], *z* = 29.11, *p* < 0.001) in the professionals' subgroup (Figure 2). Heterogeneity was high in both subgroups, with all *I* ^2^ values exceeding 90% (*p* < 0.001), indicating substantial between‐study variability.

In the managers' subgroup, the highest pooled mean was in the supportive leadership subscale (mean = 3.07, 95% CI [2.44–3.69], *z* = 9.65, *p* < 0.001), followed by the perseverant (mean = 2.57, 95% CI [1.92–3.22], *z* = 7.75, *p* < 0.001), knowledgeable (mean = 2.38, 95% CI [1.81–2.96], *z* = 8.12, *p* < 0.001), and proactive leadership (mean = 2.08, 95% CI [1.57–2.60], z = 7.92, *p* < 0.001).

In the professionals subgroup, the highest pooled mean was in the supportive leadership subscale (mean = 2.98, 95% CI [2.82–3.15], *z* = 35.71, *p* < 0.001), followed by the knowledgeable leadership (mean = 2.87, 95% CI [2.64–3.09], *z* = 25.14, *p* < 0.001), perseverant leadership (mean = 2.81, 95% CI [2.62–3.00], *z* = 29.31, *p* < 0.001), and proactive leadership (mean = 2.50, 95% CI [2.32–2.68], *z* = 27.25, *p* < 0.001). Across all subscales and subgroups, heterogeneity remained consistently high (*I* ^2^ > 90%, *p* < 0.001). Subgroup‐specific pooled mean levels of the IL subscales are presented in Table 2.

## 4 Discussion

To our knowledge, this is the first systematic review and meta‐analysis to examine pooled mean levels of IL in EBP across healthcare internationally. By synthesizing findings from 22 studies, this review shows that IL levels were consistently moderate, aligning with earlier single‐study findings despite contextual variability (Egeland et al. 2023; Finn et al. 2016; Williams et al. 2020). These results reinforce longstanding concerns about persistent gaps and developmental needs in IL capacity across healthcare systems (Cleary‐Holdforth et al. 2021; Kitson et al. 2021).

These findings of moderate IL levels are particularly significant given the increasing complexity and ongoing transformation of healthcare systems, which demand adaptive organizational practices and leadership to ensure high‐quality and equitable care (Rajit et al. 2024). As EBP is a cornerstone of informed decision‐making and quality improvement (Jordan et al. 2019; Melnyk and Fineout‐Overholt 2019), moderate IL levels may limit organizations' capacity to respond to evolving clinical demands. Because leadership is a central determinant of implementation success (Kitson et al. 2021; Nordin et al. 2022; Williams et al. 2020), inadequate IL may hinder the integration of evidence into routine practice and impede improvements in care quality and patient outcomes (Dopp et al. 2024). Strengthening IL is therefore essential not only to support EBP implementation but also to enable healthcare systems to navigate complexity and reduce inequities in care (Connor et al. 2023).

This review identifies consistent patterns in ILS subscales, with supportive leadership as the strongest and proactive leadership as the weakest across studies. The consistently low ratings for proactive leadership highlight a critical developmental need, particularly emphasized anticipatory planning and active facilitation in frameworks such as EPIS and the JBI Model (Aarons et al. 2011; Jordan et al. 2019). Development needs were evident across all ILS subscales, aligning with earlier concerns about gaps in supportive, knowledgeable, perseverant, and proactive leadership behaviors (Cleary‐Holdforth et al. 2021; Gifford et al. 2019; Kitson et al. 2021). Strengthening IL is therefore essential for achieving the organizational and individual benefits associated with EBP (Egeland et al. 2023; Sklar et al. 2024; Williams et al. 2020).

Another important finding of this review is that managers reported lower IL levels than professionals, in contrast to earlier research suggesting that managers typically rate their own IL behaviors more positively (Torres et al. 2017). This may reflect greater self‐criticism among managers due to their deeper awareness of the complexities and responsibilities of EBP implementation (Koivunen et al. 2024). This aligns with the acknowledged role of leadership in navigating implementation challenges (Li et al. 2018; Williams et al. 2020) and suggests that managers' self‐evaluations may represent a more realistic appraisal of the challenges they face. Additionally, both managers and healthcare professionals often report limited confidence in their EBP competence (e.g., Cleary‐Holdforth et al. 2021; Nwe et al. 2024), which may shape how IL behaviors are enacted and perceived.

The review highlights role‐specific differences in ILS subscales. While supportive leadership was the highest rated in both groups, professionals rated knowledgeable leadership higher than perseverant leadership, whereas managers prioritized the opposite pattern. These differences likely reflect distinct role expectations: professionals may value leaders' EBP expertise, whereas managers emphasize sustained effort in driving implementation, as highlighted in the EPIS framework (Aarons et al. 2011) and the JBI Model (Jordan et al. 2019). Such role‐specific emphases may also intersect with the documented variability in ILS scores across settings and populations (Braathu et al. 2022; Mandrou et al. 2020), and with evidence showing that nurses' EBP implementation is strongly influenced by organizational support and leadership behaviors (Mehra et al. 2025). This suggests that contextual and cultural factors may influence how IL is perceived and operationalized.

### 4.1 Limitations

While this study provides valuable insights, several limitations should be acknowledged. First, the inclusion of only English, Finnish, and Swedish studies may introduce potential language bias, and despite specialist input, errors in the search strategy cannot be fully excluded. Substantial heterogeneity was observed across the included studies, as indicated by consistently high *I* ^2^ values. This suggests considerable between‐study variability, potentially reflecting contextual, organizational, or methodological differences, and warrants caution when interpreting the pooled estimates.

## 5 Conclusions

The results of this first meta‐analytic synthesis of IL in EBP indicate that IL remains at a moderate level globally, characterized by stronger supportive leadership and weaker proactive leadership. The review also reveals clear role‐based differences, with managers reporting lower IL levels than professionals. The substantial heterogeneity across studies highlights the influence of diverse organizational and contextual factors on IL and underscores the need for tailored strategies to strengthen leadership capacity in different healthcare settings.

## 6 Linking Evidence to Action

- Strengthening IL in EBP is needed across healthcare.
- Organizations should invest in structured and targeted leadership development initiatives that foster supportive, knowledgeable, perseverant, and proactive leadership behaviors, with a particular emphasis on proactive leadership
- Leadership development should be context‐sensitive and adaptable due to substantial variability across healthcare settings.
- Role‐based differences indicate that managers especially benefit from tailored support that reflects the complexity and accountability of their leadership roles
- Further research should clarify how IL shapes implementation processes and the moderating effects of organizational structures and context. Longitudinal, intervention, and cross‐system comparative studies are needed to establish causality, evaluate leadership development programs, and inform context‐sensitive leadership interventions across various healthcare and cultural settings.

## Funding

The authors have nothing to report.

## Ethics Statement

The authors have nothing to report.

## Conflicts of Interest

The authors declare no conflicts of interest.

## Supporting information


**Table S1:** The Preferred Reporting Items for Systematic Reviews and Meta‐analysis (PRISMA) statement.
**Table S2:** Search strategies in searched databases and search results.
**Table S3:** Studies ineligible following full‐text review.
**Table S4:** Quality assessment scores of the selected studies according to Joanna Briggs Institutes analytical cross‐sectional studies.
**Table S5:** Data extraction.
**Table S6:** The effect sizes expressed as weighted mean differences and their 95% confidence intervals for the individual studies (ILS total).
**Table S7:** The effect sizes expressed as weighted mean differences and their 95% confidence intervals for the individual studies (ILS proactive).
**Table S8:** The effect sizes expressed as weighted mean differences and their 95% confidence intervals for the individual studies (ILS knowledgeable).
**Table S9:** The effect sizes expressed as weighted mean differences and their 95% confidence intervals for the individual studies (ILS supportive).
**Table S10:** The effect sizes expressed as weighted mean differences and their 95% confidence intervals for the individual studies (ILS perseverant).

## Data Availability

The data that support the findings of this study are available in Tables S1–S10.
