# Supplementary material for: Implementation Leadership in Evidence‐Based Practice in Healthcare: A Systematic Review and Meta‐Analysis
Source: Worldviews Evid Based Nurs. 2026 Aug 10;23(4):e70163. doi: 10.1111/wvn.70163 (PMC13458221; doi:10.1111/wvn.70163)
Supplement: Supplementary file 1 [file WVN-23-0-s001.docx]

Table S1. The Preferred Reporting Items for Systematic Reviews and Meta-analysis (PRISMA) statement

| **Section and Topic** | **Item #** | **Checklist item** | **Location where item is reported** |
| --- | --- | --- | --- |
| **TITLE** | | |  |
| Title | 1 | Identify the report as a systematic review. | Title page, 1 |
| **ABSTRACT** | | |  |
| Abstract | 2 | See the PRISMA 2020 for Abstracts checklist. | 1 |
| **INTRODUCTION** | | |  |
| Rationale | 3 | Describe the rationale for the review in the context of existing knowledge. | 3-4 |
| Objectives | 4 | Provide an explicit statement of the objective(s) or question(s) the review addresses. | 5 |
| **METHODS** | | |  |
| Eligibility criteria | 5 | Specify the inclusion and exclusion criteria for the review and how studies were grouped for the syntheses. | 5, Table 1. |
| Information sources | 6 | Specify all databases, registers, websites, organisations, reference lists and other sources searched or consulted to identify studies. Specify the date when each source was last searched or consulted. | 5, Figure 1. |
| Search strategy | 7 | Present the full search strategies for all databases, registers and websites, including any filters and limits used. | Table S2. |
| Selection process | 8 | Specify the methods used to decide whether a study met the inclusion criteria of the review, including how many reviewers screened each record and each report retrieved, whether they worked independently, and if applicable, details of automation tools used in the process. | 5-6 |
| Data collection process | 9 | Specify the methods used to collect data from reports, including how many reviewers collected data from each report, whether they worked independently, any processes for obtaining or confirming data from study investigators, and if applicable, details of automation tools used in the process. | 5-6 |
| Data items | 10a | List and define all outcomes for which data were sought. Specify whether all results that were compatible with each outcome domain in each study were sought (e.g. for all measures, time points, analyses), and if not, the methods used to decide which results to collect. | 6, Table S5 |
|  | 10b | List and define all other variables for which data were sought (e.g. participant and intervention characteristics, funding sources). Describe any assumptions made about any missing or unclear information. | 6 |
| Study risk of bias assessment | 11 | Specify the methods used to assess risk of bias in the included studies, including details of the tool(s) used, how many reviewers assessed each study and whether they worked independently, and if applicable, details of automation tools used in the process. | 6 |
| Effect measures | 12 | Specify for each outcome the effect measure(s) (e.g. risk ratio, mean difference) used in the synthesis or presentation of results. | 6 |
| Synthesis methods | 13a | Describe the processes used to decide which studies were eligible for each synthesis (e.g. tabulating the study intervention characteristics and comparing against the planned groups for each synthesis (item #5)). | 5, Table 1 |
|  | 13b | Describe any methods required to prepare the data for presentation or synthesis, such as handling of missing summary statistics, or data conversions. | 6 |
|  | 13c | Describe any methods used to tabulate or visually display results of individual studies and syntheses. | 6 |
|  | 13d | Describe any methods used to synthesize results and provide a rationale for the choice(s). If meta-analysis was performed, describe the model(s), method(s) to identify the presence and extent of statistical heterogeneity, and software package(s) used. | 6 |
|  | 13e | Describe any methods used to explore possible causes of heterogeneity among study results (e.g. subgroup analysis, meta-regression). | 6 |
|  | 13f | Describe any sensitivity analyses conducted to assess robustness of the synthesized results. | 6 |
| Reporting bias assessment | 14 | Describe any methods used to assess risk of bias due to missing results in a synthesis (arising from reporting biases). | 6 |
| Certainty assessment | 15 | Describe any methods used to assess certainty (or confidence) in the body of evidence for an outcome. | 6 |
| **RESULTS** | | |  |
| Study selection | 16a | Describe the results of the search and selection process, from the number of records identified in the search to the number of studies included in the review, ideally using a flow diagram. | 5-6, Figure 1 |
|  | 16b | Cite studies that might appear to meet the inclusion criteria, but which were excluded, and explain why they were excluded. | 5-6, Table S3 |
| Study characteristics | 17 | Cite each included study and present its characteristics. | 6, Table S5 |
| Risk of bias in studies | 18 | Present assessments of risk of bias for each included study. | 7-8, Table S6-S10 |
| Results of individual studies | 19 | For all outcomes, present, for each study: (a) summary statistics for each group (where appropriate) and (b) an effect estimate and its precision (e.g. confidence/credible interval), ideally using structured tables or plots. | 7-8, Figure 2, Table 2, Table S6-S10 |
| Results of syntheses | 20a | For each synthesis, briefly summarise the characteristics and risk of bias among contributing studies. | 7-8, Table S6-S10 |
|  | 20b | Present results of all statistical syntheses conducted. If meta-analysis was done, present for each the summary estimate and its precision (e.g. confidence/credible interval) and measures of statistical heterogeneity. If comparing groups, describe the direction of the effect. | 7-8, Figure 2, Table 2, Table S6-S10 |
|  | 20c | Present results of all investigations of possible causes of heterogeneity among study results. | 7-8 |
|  | 20d | Present results of all sensitivity analyses conducted to assess the robustness of the synthesized results. | 7-8, Figure 2, Table 2, Table S6-S10 |
| Reporting biases | 21 | Present assessments of risk of bias due to missing results (arising from reporting biases) for each synthesis assessed. | 7-8 |
| Certainty of evidence | 22 | Present assessments of certainty (or confidence) in the body of evidence for each outcome assessed. | Table S6-S10 |
| **DISCUSSION** | | |  |
| Discussion | 23a | Provide a general interpretation of the results in the context of other evidence. | 8-9 |
|  | 23b | Discuss any limitations of the evidence included in the review. | 9 |
|  | 23c | Discuss any limitations of the review processes used. | 9 |
|  | 23d | Discuss implications of the results for practice, policy, and future research. | 10 |
| **OTHER INFORMATION** | | |  |
| Registration and protocol | 24a | Provide registration information for the review, including register name and registration number, or state that the review was not registered. | 2 |
|  | 24b | Indicate where the review protocol can be accessed, or state that a protocol was not prepared. | 2 |
|  | 24c | Describe and explain any amendments to information provided at registration or in the protocol. | - |
| Support | 25 | Describe sources of financial or non-financial support for the review, and the role of the funders or sponsors in the review. | 10 |
| Competing interests | 26 | Declare any competing interests of review authors. | 11 |
| Availability of data, code and other materials | 27 | Report which of the following are publicly available and where they can be found: template data collection forms; data extracted from included studies; data used for all analyses; analytic code; any other materials used in the review. | 11 |

*From:* Page MJ, McKenzie JE, Bossuyt PM, Boutron I, Hoffmann TC, Mulrow CD, et al. The PRISMA 2020 statement: an updated guideline for reporting systematic reviews. BMJ 2021;372:n71. doi: 10.1136/bmj.n71. This work is licensed under CC BY 4.0. To view a copy of this license, visit <https://creativecommons.org/licenses/by/4.0/>

Table S2. Search strategies in searched databases and search results.

| Database | Keywords | Results |
| --- | --- | --- |
| Scopus | "implementation leadership" | 187 |
| Web of Science | "implementation leadership" | 159 |
| MEDLINE (Ovid) | "implementation leadership" | 143 |
| ProQuest | noft("implementation leadership") | 111 |
| CINAHL | "implementation leadership" | 77 |
| Medic | käyttöönot* AND johta* | 23 |

Searches conducted in November 2025 were limited to English, Finnish, or Swedish, and no date limitations applied.

(Source: Author's own work)

Table S3. Studies ineligible following full-text review.

1. Aranbarri, A., Stahmer, A. C., Talbott, M. R., Miller, M. E., Drahota, A., Pellecchia, M., Barber, A. B., Griffith, E. M., Morgan, E. H., & Rogers, S. J. (2021). Examining US Public Early Intervention for Toddlers With Autism: Characterizing Services and Readiness for Evidence-Based Practice Implementation. *Frontiers in Psychiatry*, 12, 786138.
   *Reason for exclusion*: Wrong population.
2. Platis, C., Delimpaltadakis, E., Stergiannis, P., Kostagiolas, P., & Intas, G. (2021). Evidence-Based Leadership: A Study of Its Application to General Hospital of the Public Health System Through the Implementation Leadership Scale. *Advances in Experimental Medicine and Biology,* 1337, 9–15.

*Reason for exclusion*: Full-text not available.

3. Spoelstra, S. L., Schueller, M., Dorn, E., & Sikorskii, A. (2022). Measuring Organizational Readiness for Change in Michigan's Home and Community-based Services Program: Instrument Adaptation and Psychometric Testing. *Home Health Care Services Quarterly*, 41(3), 255–266.

*Reason for exclusion*: Full-text not available.

4. Finn, N. K., Torres, E. M., Ehrhart, M. G., Roesch, S. C., & Aarons, G. A. (2016). Cross-Validation of the Implementation Leadership Scale (ILS) in Child Welfare Service Organizations. *Child Maltreatment*, 21(3), 250–255.

*Reason for exclusion*: Wrong setting.

5. Shuman, C. J. (2017). *Addressing the Practice Context in Evidence-Based Practice Implementation: Leadership and Climate.* Leadership & Climate, University of Michigan.

*Reason for exclusion*: Wrong study design.

6. Kujala, S., Hörhammer, I., Ervast, M., Kolann, H., & Rauhala, M. (2018). Johtamisen hyvät käytännöt sähköisten omahoitopalveluiden käyttöönotossa. *Finnish Journal of eHealth and eWelfare,*10(2–3):221–235.

*Reason for exclusion*: Wrong phenomenon of interest.

7. Aarons, G. A., & Ehrhart, M. G. (2022). Leading implementation by focusing on strategic implementation leadership. *Implementation Science*, 178-180.

*Reason for exclusion*: Wrong study design.

8. Ronquillo, C., Dahinten, V. S., Bungay, V., & Currie, L. M. (2019). The Nurse LEADership for Implementing Technologies - Mobile Health Model (Nurse LEAD-IT - mHealth). *Nursing Leadership,* 32(2), 71–84.

*Reason for exclusion*: Full-text not available.

9. Rosen, C. S., Kaplan, A. N., Nelson, D. B., La Bash, H., Chard, K. M., Eftekhari, A., Kehle-Forbes, S., Wiltsey Stirman, S., & Sayer, N. A. (2023). Implementation context and burnout among Department of Veterans Affairs psychotherapists prior to and during the COVID-19 pandemic. *Journal of Affective Disorders,* 320, 517–524.

*Reason for exclusion*: Wrong study design.

10. Reponen, E., Jokela, R., Blodgett, J. C., Rundall, T. G., Shortell, S. M., Nuutinen, M., Skants, N., Mäkijärvi, M., & Torkki, P. (2021). Validation of the Lean Healthcare Implementation Self-Assessment Instrument (LHISI) in the finnish healthcare context. *BMC Health Services Research*, 21(1), 1289.

*Reason for exclusion*: Wrong phenomenon of interest.

11. Delibaltadakis, Ε., Alikari, V., Theofilou, P., Messaropoulos, P., & Platis, C. (2020). Evidence-based nursing leadership: A study using the implementation leadership scale. *Archives of Hellenic Medicine*, 37(6), 758–764.

*Reason for exclusion:* Wrong language.

12. Egeland, K. M., Borge, R. H., Peters, N., Bækkelund, H., Braathu, N., Sklar, M., Aarons, G. A., & Skar, A. S. (2023). Individual-level associations between implementation leadership, climate, and anticipated outcomes: a time-lagged mediation analysis. *Implementation Science Communications,* 4(1), 75.

*Reason for exclusion*: Wrong study design.

13. Richter, A., Lornudd, C., von Thiele Schwarz, U., Lundmark, R., Mosson, R., Eskner Skoger, U., Hirvikoski, T., & Hasson, H. (2020). Evaluation of iLead, a generic implementation leadership intervention: mixed-method preintervention-postintervention design. *BMJ Open,* 10(1), e033227.

*Reason for exclusion*: Wrong study design.

14. Skar, A. S., Braathu, N., Peters, N., Bækkelund, H., Endsjø, M., Babaii, A., Borge, R. H., Wentzel-Larsen, T., Ehrhart, M. G., Sklar, M., Brown, C. H., Aarons, G. A., & Egeland, K. M. (2022). A stepped-wedge randomized trial investigating the effect of the Leadership and Organizational Change for Implementation (LOCI) intervention on implementation and transformational leadership, and implementation climate. *BMC Health Services Research*, 22(1), 298.

*Reason for exclusion*: Wrong study design.

15. Grove, A., Clarke, A., Currie, G., Metcalfe, A., Pope, C., & Seers, K. (2020). Advancing clinical leadership to improve the implementation of evidence-based practice in surgery: a longitudinal mixed-method study protocol. *Implementation Science,* 15(1), 104.

*Reason for exclusion:* Not a research article.

16. Sklar, M., Ehrhart, M., Roesch, S., & Aarons, G. (2022). Change in implementation leadership, climate, and provider reach for motivational interviewing: A cluster randomized trial of the leadership and organizational change for implementation (LOCI) strategy in substance use disorder treatment. *Implementation Science*, 17(suppl 1).

*Reason for exclusion:* Full-text not available.

17. Kitson, A. L., Harvey, G., Gifford, W., Hunter, S. C., Kelly, J., Cummings, G. G., Ehrenberg, A., Kislov, R., Pettersson, L., Wallin, L., & Wilson, P. (2021). How nursing leaders promote evidence-based practice implementation at point-of-care: A four-country exploratory study. *Journal of Advanced Nursing*, 77(5), 2447–2457.

*Reason for exclusion*: Wrong study design.

18. Williams, N. J., Wolk, C. B., Becker-Haimes, E. M., & Beidas, R. S. (2020). Testing a theory of strategic implementation leadership, implementation climate, and clinicians' use of evidence-based practice: a 5-year panel analysis. *Implementation Science*, 15(1), 10.

*Reason for exclusion*: Wrong study design.

19. Singh, R. R., Dolcini, M. M., Catania, J. A., & Saldana, L. (2023). Investigating Supervision and Leadership Practices to Strengthen EBP Implementation in the Child Welfare System: A Case Study Analysis. *Families in Society*, 104(4), 476-489.

*Reason for exclusion*: Wrong setting.

20. Aarons, G., Ehrhart, M., & Torres, E. (2016). Linking team level implementation leadership and implementation climate to individual level attitudes, behaviors, and implementation outcomes. *Implementation Science*, 11.

*Reason for exclusion:* Full-text not available.

21. Borge, R. H., Egeland, K. M., Aarons, G. A., Ehrhart, M. G., Sklar, M., & Skar, A. S. (2022). "Change Doesn't Happen by Itself": A Thematic Analysis of First-Level Leaders' Experiences Participating in the Leadership and Organizational Change for Implementation (LOCI) Strategy. *Administration and Policy in Mental Health*, 49(5), 785–797.

*Reason for exclusion*: Wrong study design.

22. Gifford, W., Lewis, K. B., Eldh, A. C., Fiset, V., Abdul-Fatah, T., Aberg, A. C., Thavorn, K., Graham, I. D., & Wallin, L. (2019). Feasibility and usefulness of a leadership intervention to implement evidence-based falls prevention practices in residential care in Canada. *Pilot and Feasibility Studies*, 5, 103.

*Reason for exclusion*: Wrong study design.

23. Hu, J., Gifford, W., Ruan, H., Harrison, D., Li, Q., Ehrhart, M. G., & Aarons, G. A. (2019). Translation and linguistic validation of the implementation leadership scale in Chinese nursing context. *Journal of Nursing Management*, 27(5), 1030–1038.

*Reason for exclusion*: Wrong study design.

24. Richter, A., von Thiele Schwarz, U., Lornudd, C., Lundmark, R., Mosson, R., & Hasson, H. (2016). iLead-a transformational leadership intervention to train healthcare managers' implementation leadership. *Implementation Science*, 11, 108.

*Reason for exclusion*: Wrong study design.

25. Byeon, Y. V., Lau, A. S., Lind, T., Hamilton, A. B., & Brookman-Frazee, L. (2022). Organizational factors associated with community therapists' self-efficacy in EBP delivery: The interplay between sustainment leadership, sustainment climate, and psychological safety. *Implementation Research and Practice*, 3, 26334895221110263.

*Reason for exclusion*: Wrong phenomenon of interest.

26. Ge, L., Zhang, Y., Hu, Y., Chen, X., Liang, Y., & Wang, A. (2022). Correlation between evidence‑based practice implementation leadership of nursing managers and evidence‑based nursing competencies of clinical nurses with master's degrees. *Chinese Nursing Research*, 36(14): 2476-2480.

*Reason for exclusion*: Wrong language.

27. Torres, E. M., Ehrhart, M. G., Beidas, R. S., Farahnak, L. R., Finn, N. K., & Aarons, G. A. (2018). Validation of the Implementation Leadership Scale (ILS) with Supervisors' Self-Ratings. *Community Mental Health Journal*, 54(1), 49–53.

*Reason for exclusion*: Wrong phenomenon of interest.

28. Proctor, E., Ramsey, A. T., Brown, M. T., Malone, S., Hooley, C., & McKay, V. (2019). Training in Implementation Practice Leadership (TRIPLE): evaluation of a novel practice change strategy in behavioral health organizations. *Implementation Science*, 14(1), 66.

*Reason for exclusion*: Wrong study design.

29. Wakefield, B. J., Drwal, K., Paez, M., Grover, S., Franciscus, C., Reisinger, H. S., Kaboli, P. J., & El Accaoui, R. (2019). Creating and disseminating a home-based cardiac rehabilitation program: experience from the Veterans Health Administration. *BMC Cardiovascular Disorders*, 19(1), 242.

*Reason for exclusion*: Wrong phenomenon of interest.

30. Janssen, D.G. (2011). *The effect of nursing leadership and teaching methodologies on the level of adoption of an electronic health record (EHR) implementation*. Southeastern Louisiana University, (Ed.D.):156–156.

*Reason for exclusion:* Not a research article.

31. Gifford, W., Graham, I. D., Ehrhart, M. G., Davies, B. L., & Aarons, G. A. (2017). Ottawa Model of Implementation Leadership and Implementation Leadership Scale: mapping concepts for developing and evaluating theory-based leadership interventions. *Journal of Healthcare Leadership,* 9, 15–23.

*Reason for exclusion*: Wrong study design.

32. Nordin, H., Rørtveit, K., Mathisen, G. E., Joa, I., Johannessen, J. O., Ruud, T., & Hartveit, M. (2022). Frontline leadership for implementing clinical guidelines in Norwegian mental health services: a qualitative study. *Journal of Health Organization and Management*, ahead-of-print(ahead-of-print), 10.1108/JHOM-08-2021-0286.

*Reason for exclusion*: Wrong study design.

33. Aarons, G. A., Ehrhart, M. G., Farahnak, L. R., & Hurlburt, M. S. (2015). Leadership and organizational change for implementation (LOCI): a randomized mixed method pilot study of a leadership and organization development intervention for evidence-based practice implementation. *Implementation Science*, 10, 11.

*Reason for exclusion*: Wrong study design.

34. Heinemann, A. W., Nitsch, K. P., Ehrlich-Jones, L., Malamut, L., Semik, P., Srdanovic, N., Kocherginsky, M., & Hobbs, S. (2019). Effects of an Implementation Intervention to Promote Use of Patient-Reported Outcome Measures on Clinicians' Perceptions of Evidence-Based Practice, Implementation Leadership, and Team Functioning. *The Journal of Continuing Education in the Health Professions*, 39(2), 103–111.

*Reason for exclusion*: Wrong study design.

35. Ehrhart, M. G., Torres, E. M., Green, A. E., Trott, E. M., Willging, C. E., Moullin, J. C., & Aarons, G. A. (2018). Leading for the long haul: a mixed-method evaluation of the Sustainment Leadership Scale (SLS). *Implementation Science*, 13(1), 17.

*Reason for exclusion*: Wrong population.

36. Vasilopoulou, G., Tsatsou, I., Konstantinidis, T., & Roka, V. (2023). Relationship between job satisfaction of nursing staff and leadership that supports and promotes evidence‑based practice. *Nosileftiki*, 62(1): 105–116.

*Reason for exclusion*: Wrong language.

37. Ronquillo, C., Dahinten, S., Bungay, V., & Currie, L. (2018). The MOBile-Health and Implementation Leadership Evaluation (MOBILE) Nurse Study: Developing a Testable Conceptual Model. *Studies in Health Technology and Informatics*, 250, 143.

*Reason for exclusion:* Full-text not available.

*38.* Guerrero, E. G., Padwa, H., Fenwick, K., Harris, L. M., & Aarons, G. A. (2016). Identifying

and ranking implicit leadership strategies to promote evidence-based practice implementation in addiction health services. *Implementation Science*, 11, 69.

*Reason for exclusion:* Wrong study design.

*39.* Egeland, K.M., Sklar, M., Aarons, G.A., Ehrhart, M.G., Skar, A-M.S. & Borge, R.H. (2024).

Symphony of Success: Leader-Practitioner Reciprocity during Evidence-Based Practice Implementation. *Administration and Policy in Mental Health and Mental Health Services Research,* 51(6): 988–996.

*Reason for exclusion:* Wrong study design.

40. Gago, C., De Leon, E., Mandal S., de la Calle, F, Garcia, M., Colella, D., Dapkins, I. &

Schoenthaler, A. (2024). "Hypertension is such a difficult disease to manage": federally qualified health center staff- and leadership-perceived readiness to implement a technology-facilitated team-based hypertension model. *Implementation Science Communication*, 5(1), 49.

*Reason for exclusion:* Wrong study design.

41*.* Mosson, R., von Thiele Schwarz, U., Hasson, H., Lundmark, R., & Richter, A. (2018). How

Do iLead? Validation of a scale measuring active and passive implementation leadership in Swedish healthcare. *BMJ Open,* 8(6), e021992. <https://doi.org/10.1136/bmjopen-2018-021992>

*Reason for exclusion*: Wrong phenomenon of interest.

42. Bartels, S. M., Dang, H. L. T., Chen, J. S., Nong, H. T. T., Cao, V. A. T., Tran, H. V.,

Sripaipan, T., Powell, B. J., Barrington, C., Reyes, L. M., Giang, L. M., Phan, H. T. T., Miller, W. C., & Go, V. F. (2025). A Mixed Methods Assessment of the Effect of Organizational Characteristics on Intervention Scale-Up Success in 42 HIV Testing Clinics in Vietnam. *Journal of Evaluation in Clinical Practice,* 31(5), e70243. <https://doi.org/10.1111/jep.70243>

*Reason for exclusion:* Wrong study design.

43. Powell, B. J., Mandell, D. S., Hadley, T. R., Rubin, R. M., Evans, A. C., Hurford, M. O., &

Beidas, R. S. (2017). Are general and strategic measures of organizational context and leadership associated with knowledge and attitudes toward evidence-based practices in public behavioral health settings? A cross-sectional observational study. *Implementation Science,* 12(1), 64. <https://doi.org/10.1186/s13012-017-0593-9>

*Reason for exclusion*: Wrong phenomenon of interest.

44. Guerrero, E. G., Frimpong, J., Kong, Y., Fenwick, K., & Aarons, G. A. (2020). Advancing

theory on the multilevel role of leadership in the implementation of evidence-based health care practices. *Health Care Management Review*, 45(2), 151–161. <https://doi.org/10.1097/HMR.0000000000000213>

*Reason for exclusion*: Wrong population.

45. Hu, S., Liu, S., Li, X., Zhao, J., Chen, J., Chen, W., & Hu, J. (2025). Organizational

evidence-based practice culture, implementation leadership, and nurses: A bidirectional mediation model. *International Nursing Review*, 72(2), e13054. <https://doi.org/10.1111/inr.13054>

*Reason for exclusion*: Wrong phenomenon of interest.

46. Mosson, R., von Thiele Schwarz, U., Hasson, H., Lundmark, R., & Richter, A. (2018).

How do iLead? Validation of a scale measuring active and passive implementation leadership in Swedish healthcare. *BMJ Open*, 8(6), e021992. <https://doi.org/10.1136/bmjopen-2018-021992>

*Reason for exclusion*: Wrong phenomenon of interest.

(Source: Authors own work)

Table S4. Quality assessment scores of the selected studies according to Joanna Briggs Institutes analytical cross-sectional studies.

| Study | Q1 | Q2 | Q3 | Q4 | Q5 | Q6 | Q7 | Q8 | Total |
| --- | --- | --- | --- | --- | --- | --- | --- | --- | --- |
| Aarons et al. 2014 | Y | Y | Y | Y | Y | N | Y | Y | 7/8 |
| Aarons et al. 2016 | Y | Y | Y | Y | Y | N | Y | Y | 7/8 |
| Aarons et al. 2017 | Y | Y | Y | Y | Y | Y | Y | Y | 8/8 |
| Boehm et al. 2022 | Y | Y | Y | Y | Y | Y | Y | Y | 8/8 |
| Borge et al. 2021 | Y | Y | Y | Y | Y | N | Y | Y | 7/8 |
| Braathu et al. 2022 | Y | Y | Y | Y | Y | N | Y | Y | 7/8 |
| Hsu et al. 2025 | Y | Y | Y | Y | Y | N | Y | Y | 7/8 |
| Hu et al. 2021 | Y | Y | Y | Y | Y | N | Y | Y | 7/8 |
| Kano et al. 2025 | Y | Y | Y | Y | N | N | Y | Y | 6/8 |
| J. Li et al. 2025 | Y | Y | Y | Y | Y | Y | Y | Y | 8/8 |
| L. Li et al. 2025 | Y | Y | Y | Y | Y | Y | Y | Y | 8/8 |
| Llarena et al. 2023 | Y | Y | Y | Y | Y | N | Y | Y | 7/8 |
| Mandrou et al. 2020 | Y | Y | Y | Y | Y | N | Y | Y | 7/8 |
| Ronquillo et al. 2023 | Y | Y | Y | Y | Y | Y | Y | Y | 8/8 |
| Saiki et al. 2023 | Y | Y | Y | Y | Y | Y | Y | Y | 8/8 |
| Saiki et al. 2025 | Y | Y | Y | Y | Y | Y | Y | Y | 8/8 |
| Shuman et al. 2018 | Y | Y | Y | Y | Y | Y | Y | Y | 8/8 |
| Shuman et al. 2019 | Y | Y | Y | Y | Y | Y | Y | Y | 8/8 |
| Shuman et al. 2020 | Y | Y | Y | Y | Y | N | Y | Y | 7/8 |
| Shuman et al. 2023 | Y | Y | Y | Y | Y | Y | Y | Y | 7/8 |
| Söling et al. 2022 | Y | Y | Y | Y | Y | N | Y | Y | 7/8 |
| Yıldız 2024 | Y | Y | Y | Y | Y | N | Y | Y | 7/8 |

*Note:* Q1: Were the criteria for inclusion in the sample clearly defined? Q2: Were the study subjects and the setting described in detail? Q3: Was the exposure measured in a valid and reliable way? Q4: Were objective, standard criteria used for measurement of the condition? Q5: Were confounding factors identified? Q6: Were strategies to deal with confounding factors stated? Q7: Were the outcomes measured in a valid and reliable way? Q8: Was appropriate statistical analysis used?

Abbreviations: *Y*, Yes; *N*, No; *U*, Unclear; NA, Not applicable.

(Source: Authors own work)

Table S5. Data extraction.

| Authors (year) Country of origin | Participants (sample size) | Data collection and data analysis | Measures | Key findings |
| --- | --- | --- | --- | --- |
| Aarons et al. (2014)  United States | Mental health clinicians (n = 459) from 93 outpatient programs. | Data collection:  Online and in-person paper-and-pencil surveys.  Data analysis:  Means, SDs. | ILS | ILS mean scores were 2.42 (SD 1.12) on the total ILS scale 2.12 (SD 1.25) on the proactive leadership subscale, 2.56 (SD 1.18) on the knowledgeable leadership subscale, 2.63 (SD 1.15) on the supportive leadership subscale, and 2.36 (SD 1.25) on the perseverant leadership subscale. |
| Aarons et al. (2016)  United States | Service providers (n = 323) from 72 workgroups across three alcohol and other drug treatment agencies. | Data collection:  Online and in-person surveys.  Data analysis:  Means, SDs. | ILS | ILS mean scores were 2.77 (SD 0.97) on the total ILS scale, 2.51 (SD 1.13) on the proactive leadership subscale, 2.95 (SD 1.00) on the knowledgeable leadership subscale, 2.90 (SD 1.02) on the supportive leadership subscale, and 2.72 (SD 1.06) on the perseverant leadership subscale. |
| Aarons et al. (2017)  United States | Leaders of public mental health teams (n = 80) and service providers (n = 513) from 31 mental health service organisations. | Data collection:  Online and in-person paper-and-pencil surveys.  Data analysis:  Means, SDs. | ILS | ILS mean scores were 2.42 (SD 0.79) for providers and 2.39 (SD 0.92) for leaders. |
| Boehm et al. (2022)  United States | Nurses (n = 86) from three ICUs in an academic medical center and two ICUs in a regional medical center. | Data collection:  Quantitative survey.  Data analysis:  Medians. | ILS | ILS median scores were 3.6 on the total ILS scale, 3.0 on the proactive leadership subscale, 4.0 on the knowledgeable leadership subscale, 3.7 on the supportive leadership subscale, and 3.7 on the perseverant leadership subscale. |
| Borge et al. (2021)  Norway | Leaders (n = 42) and practitioners (n = 152) in 43 mental health clinics. | Data collection:  Online survey.  Data analysis:  Means, SDs. | ILS | ILS mean scores were 2.65 (SD .79) on the total ILS scale, 2.29 (SD .95) on the proactive leadership subscale, 2.29 (SD 1.04) on the knowledgeable leadership subscale, 3.19 (SD .74) on the supportive leadership subscale, and 2.82 (SD .88) on the perseverant leadership subscale. |
| Braathu et al. (2022)  Norway | Therapists (n = 795) worked at mental health clinics for children (n = 22) and adults (n = 25). | Data collection:  Questionnaire.  Data analysis:  Means, SDs. | ILS | ILS mean scores were 2.39 (SD 0.88) on the total ILS scale, 2.05 (SD 1.01) on the proactive leadership subscale, 2.21 (SD 1.10) on the knowledgeable leadership subscale, 2.81 (SD 0.96) on the supportive leadership subscale, and 2.49 (SD 0.96) on the perseverant leadership subscale. |
| Hsu et al. (2025)  United States | Leaders (n = 21) and nurses (n = 470) from 17 labour, postpartum, pediatric, and neonatal units in four hospitals. | Data collection:  Electronic survey.  Data analysis:  Means, SDs. | ILS | ILS mean scores were 2.41 (SD 1.01) for staff nurses and 2.98 (SD = 0.88) for leaders.  ILS mean scores for staff nurses were 2.25 (SD 1.01) and 2.49 (SD 1.01) for unit leaders on the proactive leadership subscale; 2.47 (SD 1.01) for staff nurses and 2.90 (SD 0.67) for unit leader on the knowledgeable leadership subscale; 2.54 (SD 1.00) for staff nurses and 3.62 (SD 0.58) for unit leaders on the supportive leadership subscale; 2.38 (SD 0.99) for staff nurses and 2.89 (SD 0.79) for unit leaders on the perseverant leadership subscale. |
| Hu et al. (2021)  China | Nurses (n = 234) from 35 clinical units in a general tertiary hospital. | Data collection:  Online survey.  Data analysis:  means, SDs. | ILS | ILS mean scores were 2.57 (SD 0.87) on the total ILS scale, 2.38 (SD 0.93) on the proactive leadership subscale, 2.52 (SD 0.99) on the knowledgeable leadership subscale, 2.87 (SD 0.94) on the supportive leadership subscale, and 2.52 (SD 1.02) on the perseverant leadership subscale. |
| Kano et al. (2025)  United States | Nurses (n = 59, front-end staff (n = 20), and Doctors of Medicine and registered nurses (n = 13) in the University of New Mexico Comprehensive Cancer Center. | Data collection: Online survey.  Data analysis: means, SDs. | ILS | ILS mean scores were 2.88 (SD 1.25) on the total ILS scale, 2.46 (SD 1.37) on the proactive leadership subscale, 2.86 (SD 1.35) on the knowledgeable leadership subscale, 3.08 (SD 1.30) on the supportive leadership subscale, and 3.11 (SD 1.29) on the perseverant leadership subscale. |
| L. Li et al.  (2025)  China | Clinical nurses (n = 2124) from 17 secondary and tertiary hospitals. | Data collection: Online survey  Data analysis: means, SDs. | ILS | ILS mean scores were 2.88 (SD 0.74) on the total ILS scale, 2.54 (SD 0.98) on the proactive leadership subscale, 2.84 (SD 0.82) on the knowledgeable leadership subscale, 3.13 (SD 0.76) on the supportive leadership subscale, and 3.00 (SD 0.78) on the perseverant leadership subscale. |
| J. Li et al.  (2025)  China | Clinical nurses (n = 1104) from a tertiary A-level general hospital. | Data collection: Online survey.  Data analysis: means, SDs. | ILS | ILS mean scores were 3.11 (SD 0.81) on the total ILS scale, 3.02 (SD 0.85) on the proactive leadership subscale, 3.08 (SD 0.86) on the knowledgeable leadership subscale, 3.18 (SD 0.83) on the supportive leadership subscale, and 3.16 (SD 0.86) on the perseverant leadership subscale. |
| Llarena et al. (2023)  Spain | Healthcare professionals (family physicians, paediatricians, and nurses) (n = 144) in 13 integrated healthcare organisations. | Data collection:  Online survey.  Data analysis:  means, SDs. | ILS | ILS mean scores were 3.34 (SD 0.58) on the total ILS scale, 3.20 (SD 0.69) on the proactive leadership subscale, 3.43 (SD 0.64) on the knowledgeable leadership subscale, 3.44 (SD 0.68) on the supportive leadership subscale, and 3.30 (SD 0.71) on the perseverant leadership subscale. |
| Mandrou et al. (2020)  Greece | Nurses and midwives (n = 143) in a private general obstetrics and gynaecology hospital. | Data collection:  Questionnaire.  Data analysis:  Means, SDs, Cronbach alphas, Pearson correlations. | ILS | ILS mean scores were 3.19 (SD 0.56) on the total ILS scale, 3.07 (SD 0.56) on the proactive leadership subscale, 3.39 (SD 0.57) on the knowledgeable leadership subscale, 3.16 (SD 0.80) on the supportive leadership subscale, and 3.18 (SD 0.66) on the perseverant leadership subscale. |
|  |  |  |  |  |
| Ronquillo et al. (2023)  Canada | Registered nurses (n = 288) from various settings. | Data collection:  Web-based survey.  Data analysis:  Means, SDs. | ILS | ILS mean scores were 2.13 (SD 1.05). |
| Saiki et al. (2023)  Japan | Nurses (n = 484) and nurse managers (n=67) working at three university hospitals. | Data collection:  Online survey.  Data analysis:  Means, SDs. | ILS | ILS mean scores for nurses were 1.76 (SD 0.98) and 1.54 (SD = 0.81) for nurse managers on the total ILS scale.  ILS mean scores for nurses were 1.57 (SD 1.06) and 1.33 (1.04) for nurse managers on the proactive leadership subscale; 1.93 (SD 1.12) for staff nurses and 1.54 (SD 0.91) for nurse managers on the knowledgeable leadership subscale; 2.00 (SD 1.15) for staff nurses and 2.15 (SD 0.94) for nurse managers on the supportive leadership subscale; 1.79 (SD 1.13) for staff nurses and 1.58 (SD 0.94) for nurse managers on the perseverant leadership subscale. |
| Saiki et al. (2025)  Japan | Nurses (n = 467 from 84 hospital wards. | Data collection:  A secondary analysis of data collected from a cross-sectional study (Saiki et al., 2023).  Data analysis:  Means, SDs. | ILS | ILS mean scores were 1.77 (SD 0.96). |
| Shuman et al. (2018)  United States | Nurses (n = 264) from 22 units in seven acute care community hospitals. | Data collection:  Electronic questionnaire.  Data analysis:  Means, SDs. | ILS | ILS mean scores for nurses were 2.84 (SD = 0.77). |
| Shuman et al. (2019)  United States | Nurse Managers (n = 23) and registered nurses (n = 287) from 24 adult medical-surgical units. | Data collection:  Electronic questionnaire.  Data analysis:  Means, SDs. | ILS | ILS mean scores for nurses were 2.88 (SD 0.78) and 2.73 (SD = 0.46) for managers on the total ILS scale.  ILS mean scores for nurses were 2.67 (SD 0.87) and 2.25 (0.70) for managers on the proactive leadership subscale; 2.99 (SD 0.80) for nurses and 2.54 (SD 0.53) for managers on the knowledgeable leadership subscale; 3.03 (SD 0.80) for nurses and 3.23 (SD 0.54) for managers on the supportive leadership subscale; 2.84 (SD 0.84) for nurses and 2.88 (SD 0.50) for managers on the perseverant leadership subscale. |
| Shuman et al. (2020)  United States | Sample 1: Registered nurses (n = 200) from 78 units in four hospitals.  Sample 2: Registered nurses (n = 284) from 24 units in seven hospitals. | Data collection:  Electronic questionnaires.  Data analysis:  means, SDs. | ILS | ILS mean scores for sample 1 nurses were 2.61 (SD 0.90) and 2.88 (SD = 0.78) for sample 2 nurses on the total ILS scale.  ILS mean scores for sample 1 nurses were 2.31 (SD 1.05) and 2.67 (SD 0.87) for sample 2 nurses on the proactive leadership subscale; 2.70 (SD 0.95) for sample 1 nurses and 2.99 (SD 0.80) for sample 2 nurses on the knowledgeable leadership subscale; 2.75 (SD 0.92) for sample 1 nurses and 3.03 (SD 0.80) for sample 2 nurses on the supportive leadership subscale; 2.68 (SD 0.97) for sample 1 nurses and 2.84 (SD 0.84) for sample 2 nurses on the perseverant leadership subscale. |
| Shuman et al. (2023)  United States | Nurse managers (n = 22) and staff nurses (n = 261) from 22 different units in seven hospitals. | Data collection:  Web-based survey.  Data analysis:  Means, SDs. | ILS | ILS mean scores for nurses were 2.79 (SD 0.52) and 2.77 (SD = 0.40) for managers on the total ILS scale.  ILS mean scores for nurses were 2.57 (SD 0.50) and 2.30 (SD 0.65) for managers on the proactive leadership subscale; 2.90 (SD 0.54) for nurses and 2.57 (SD 0.50) for managers on the knowledgeable leadership subscale; 2.97 (SD 0.49) for nurses and 3.27 (SD 0.51) for managers on the supportive leadership subscale; 2.74 (SD 0.56) for nurses and 2.93 (SD 0.43) for managers on the perseverant leadership subscale. |
| Söling et al. (2022)  German | Primary care physicians (n = 198) of outpatient care. | Data collection:  Electronic questionnaire.  Data analysis:  Means, SDs. | ILS | ILS mean scores were 3.07 (SD 0.74) on the total ILS scale, 2.23 (SD 0.96) on the proactive leadership subscale, 3.54 (SD 0.92) on the knowledgeable leadership subscale, 3.34 (SD 1.22) on the supportive leadership subscale, and 3.08 (SD 0.97) on the perseverant leadership subscale. |
| Yıldız (2024)  Türkiye | Nurses (n = 343) at a training and research hospital. | Data collection: Questionnaires.  Data analysis:  Means, SDs. | ILS | ILS mean scores were 2.77 (SD 0.57) on the total ILS scale, 2.60 (SD 0.65) on the proactive leadership subscale, 2.81 (SD 0.87) on the knowledgeable leadership subscale, 2.97 (SD 0.80) on the supportive leadership subscale, and 2.69 (SD 0.74) on the perseverant leadership subscale. |

ILS = Implementation leadership scale

(Source: Authors own work)

Table S6. The effect sizes expressed as weighted mean differences and their 95% confidence intervals for the individual studies (ILS total)

|  | Study | Effect Size | Std. Error | Z | Sig. (2-tailed) | 95% Confidence Interval | | Weight | Weight (%) |
| --- | --- | --- | --- | --- | --- | --- | --- | --- | --- |
|  |  |  |  |  |  | Lower | Upper |  |  |
| Managers | Aarons et al. 2017 | 2.390 | 0.1029 | 23.236 | < 0.001 | 2.188 | 2.592 | 4.515 | 3.5 |
|  | Hsu et al.  2025 | 2.980 | 0.1920 | 15.518 | < 0.001 | 2.604 | 3.356 | 4.036 | 3.1 |
|  | Saiki et al. 2023 | 1.540 | 0.0990 | 15.562 | < 0.001 | 1.346 | 1.734 | 4.531 | 3.5 |
|  | Shuman et al. 2019 | 2.730 | 0.0959 | 28.462 | < 0.001 | 2.542 | 2.918 | 4.543 | 3.5 |
|  | Shuman et al. 2023 | 2.770 | 0.0853 | 32.481 | < 0.001 | 2.603 | 2.937 | 4.583 | 3.5 |
| Professionals | Aarons et al. 2014 | 2.420 | 0.0523 | 46.292 | < 0.001 | 2.318 | 2.522 | 4.681 | 3.6 |
|  | Aarons et al. 2016 | 2.770 | 0.0540 | 51.323 | < 0.001 | 2.664 | 2.876 | 4.677 | 3.6 |
|  | Aarons et al. 2017 | 2.420 | 0.0324 | 74.596 | < 0.001 | 2.356 | 2.484 | 4.718 | 3.6 |
|  | Boehm et al. 2022 | 3.600 | 0.0960 | 37.511 | < 0.001 | 3.412 | 3.788 | 4.543 | 3.5 |
|  | Borge et al. 2021 | 2.650 | 0.0641 | 41.356 | < 0.001 | 2.524 | 2.776 | 4,651 | 3.6 |
|  | Braathu et al. 2022 | 2.390 | 0.0312 | 76.577 | < 0.001 | 2.329 | 2.451 | 4.719 | 3.6 |
|  | Hsu et al.  2025 | 2.410 | 0.0478 | 50.392 | < 0.001 | 2.316 | 2.504 | 4.690 | 3.6 |
|  | Hu et al.  2021 | 2.570 | 0.0569 | 45.188 | < 0.001 | 2.459 | 2.681 | 4.670 | 3.6 |
|  | Kano et al. 2025 | 2.880 | 0.1303 | 22.099 | < 0.001 | 2.625 | 3.135 | 4.388 | 3.4 |
|  | J. Li et al.  2025 | 3.110 | 0.0244 | 127.573 | < 0.001 | 3.062 | 3.158 | 4.728 | 3.6 |
|  | L. Li et al. 2025 | 2.880 | 0.0161 | 179.365 | < 0.001 | 2.849 | 2.911 | 4.735 | 3.7 |
|  | Llarena et al. 2023 | 3.340 | 0.0483 | 69.103 | < 0.001 | 3.245 | 3.435 | 4.689 | 3.6 |
|  | Mandrou et al. 2020 | 3.190 | 0.0468 | 68.119 | < 0.001 | 3.098 | 3.282 | 4.692 | 3.6 |
|  | Ronquillo et al. 2023 | 2.130 | 0.0619 | 34.426 | < 0.001 | 2.009 | 2.251 | 4.657 | 3.6 |
|  | Saiki et al. 2023 | 1.760 | 0.0445 | 39.510 | < 0.001 | 1.673 | 1.847 | 4.697 | 3.6 |
|  | Shuman et al. 2018 | 2.840 | 0.0474 | 59.928 | < 0.001 | 2.747 | 2.933 | 4.691 | 3.6 |
|  | Shuman et al. 2019 | 2.880 | 0.0460 | 62.552 | < 0.001 | 2.790 | 2.970 | 4.694 | 3.6 |
|  | Shuman et al. 2020 (1) | 2.610 | 0.0636 | 41.012 | < 0.001 | 2.485 | 2.735 | 4.652 | 3.6 |
|  | Shuman et al. 2020 (2) | 2.880 | 0.0516 | 55.787 | < 0.001 | 2.779 | 2.981 | 4.682 | 3.6 |
|  | Shuman et al. 2023 | 2.790 | 0.0322 | 86.680 | < 0.001 | 2.727 | 2.853 | 4.718 | 3.6 |
|  | Söling et al. 2022 | 3.070 | 0.0526 | 58.377 | < 0.001 | 2.967 | 3.173 | 4.680 | 3.6 |
|  | Yıldız 2024 | 2.770 | 0.0308 | 90.002 | < 0.001 | 2.710 | 2.830 | 4.720 | 3.6 |

(Source: Authors own work)

Table S7. The effect sizes expressed as weighted mean differences and their 95% confidence intervals for the individual studies (ILS proactive).

|  | Study | Effect Size | Std. Error | Z | Sig. (2-tailed) | 95% Confidence Interval | | Weight | Weight (%) |
| --- | --- | --- | --- | --- | --- | --- | --- | --- | --- |
|  |  |  |  |  |  | Lower | Upper |  |  |
| Managers | Hsu et al.  2024 | 2.490 | 0.2204 | 11.298 | < 0.001 | 2.058 | 2.922 | 4.294 | 3.6 |
|  | Saiki et al. 2023 | 1.330 | 0.1271 | 10.468 | < 0.001 | 1.081 | 1.579 | 4.989 | 4.2 |
|  | Shuman et al. 2019 | 2.250 | 0.1460 | 15.415 | < 0.001 | 1.964 | 2.536 | 4.864 | 4.1 |
|  | Shuman et al. 2023 | 2.300 | 0.1386 | 16.597 | < 0.001 | 2.028 | 2.572 | 4.914 | 4.1 |
| Professionals | Aarons et al. 2014 | 2.120 | 0.583 | 36.336 | < 0.001 | 2.006 | 2.234 | 5.328 | 4.4 |
|  | Aarons et al. 2016 | 2.510 | 0.0629 | 39.921 | < 0.001 | 2.387 | 2.633 | 5.312 | 4.4 |
|  | Boehm et al. 2022 | 3.000 | 0.1359 | 22.080 | < 0.001 | 2.734 | 3.266 | 4.932 | 4.1 |
|  | Borge et al. 2021 | 2.290 | 0.0771 | 29.719 | < 0.001 | 2.139 | 2.441 | 5.257 | 4.4 |
|  | Braathu et al. 2022 | 2.050 | 0.0358 | 57.229 | < 0.001 | 1.980 | 2.120 | 5.389 | 4.5 |
|  | Hsu et al.  2025 | 2.250 | 0.0478 | 47.047 | < 0.001 | 2.156 | 2.344 | 5.360 | 4.5 |
|  | Hu et al.  2021 | 2.380 | 0.0608 | 39.147 | < 0.001 | 2.261 | 2.499 | 5.319 | 4.4 |
|  | Kano et al. 2025 | 2.460 | 0.1428 | 17.223 | < 0.001 | 2.180 | 2.740 | 4.885 | 4.1 |
|  | J. Li et al.  2025 | 3.020 | 0.0256 | 118.052 | < 0.001 | 2.970 | 3.070 | 5.407 | 4.5 |
|  | L. Li et al. 2025 | 2.540 | 0.0213 | 119.450 | < 0.001 | 2.498 | 2.582 | 5.413 | 4.5 |
|  | Llarena et al. 2023 | 3.200 | 0.0575 | 55.652 | < 0.001 | 3.087 | 3.313 | 5.331 | 4.5 |
|  | Mandrou et al. 2020 | 3.070 | 0.0468 | 65.557 | < 0.001 | 2.978 | 3.162 | 5.362 | 4.5 |
|  | Saiki et al. 2023 | 1.570 | 0.0482 | 32.585 | < 0.001 | 1.476 | 1.664 | 5.359 | 4.5 |
|  | Shuman et al. 2019 | 2.670 | 0.0514 | 51.992 | < 0.001 | 2.569 | 2.771 | 5.350 | 4.5 |
|  | Shuman et al. 2020 (1) | 2.310 | 0.0742 | 31.113 | < 0.001 | 2.164 | 2.456 | 5.269 | 4.5 |
|  | Shuman et al. 2020 (2) | 2.670 | 0.0516 | 51.719 | < 0.001 | 2.569 | 2.771 | 5.349 | 4.5 |
|  | Shuman et al. 2023 | 2.570 | 0.0309 | 83.039 | < 0.001 | 2.509 | 2.631 | 5.398 | 4.5 |
|  | Söling et al. 2022 | 2.230 | 0.0682 | 32.686 | < 0.001 | 2.096 | 2.364 | 5.292 | 4.5 |
|  | Yıldız 2024 | 2.600 | 0.0351 | 74.081 | < 0.001 | 2.531 | 2.669 | 5.390 | 4.5 |

(Source: Authors own work)

Table S8. The effect sizes expressed as weighted mean differences and their 95% confidence intervals for the individual studies (ILS knowledgeable).

|  | Study | Effect Size | Std. Error | Z | Sig. (2-tailed) | 95% Confidence Interval | | Weight | Weight (%) |
| --- | --- | --- | --- | --- | --- | --- | --- | --- | --- |
|  |  |  |  |  |  | Lower | Upper |  |  |
| Managers | Hsu et al.  2024 | 2.900 | 0.1462 | 19.835 | < 0.001 | 2.613 | 3.187 | 3.350 | 4.1 |
|  | Saiki et al. 2023 | 1.540 | 0.1112 | 13.852 | < 0.001 | 1.322 | 1.758 | 3.454 | 4.2 |
|  | Shuman et al. 2019 | 2.540 | 0.1105 | 22.984 | < 0.001 | 2.323 | 2.757 | 3.456 | 4.2 |
|  | Shuman et al. 2023 | 2.570 | 0.1066 | 24.109 | < 0.001 | 2.361 | 2.779 | 3.466 | 4.3 |
| Professionals | Aarons et al. 2014 | 2.560 | 0.0551 | 46.480 | < 0.001 | 2.452 | 2.668 | 3.569 | 4.4 |
|  | Aarons et al. 2016 | 2.950 | 0.0556 | 53.018 | < 0.001 | 2.841 | 3.059 | 3.568 | 4.4 |
|  | Boehm et al. 2022 | 4.000 | 0.0798 | 50.128 | < 0.001 | 3.844 | 4.156 | 3.527 | 4.3 |
|  | Borge et al. 2021 | 2.290 | 0.0844 | 27.147 | < 0.001 | 2.125 | 2.455 | 3.518 | 4.3 |
|  | Braathu et al. 2022 | 2.210 | 0.0390 | 56.648 | < 0.001 | 2.134 | 2.286 | 3.588 | 4.4 |
|  | Hsu et al.  2025 | 2.470 | 0.0478 | 51.647 | < 0.001 | 2.376 | 2.564 | 3.578 | 4.4 |
|  | Hu et al.  2021 | 2.520 | 0.0647 | 38.938 | < 0.001 | 2.393 | 2.647 | 3.554 | 4.4 |
|  | Kano et al. 2025 | 2.860 | 0.1407 | 20.320 | < 0.001 | 2.584 | 3.136 | 3.367 | 4.1 |
|  | J. Li et al.  2025 | 3.080 | 0.0259 | 118.997 | < 0.001 | 3.029 | 3.131 | 3.599 | 4.4 |
|  | L. Li et al. 2025 | 2.840 | 0.0178 | 159.618 | < 0.001 | 2.805 | 2.875 | 3.604 | 4.4 |
|  | Llarena et al. 2023 | 3.430 | 0.0533 | 64.313 | < 0.001 | 3.325 | 3.535 | 3.571 | 4.4 |
|  | Mandrou et al. 2020 | 3.390 | 0.0477 | 71.120 | < 0.001 | 3.297 | 3.483 | 3.578 | 4.4 |
|  | Saiki et al. 2023 | 1.930 | 0.0509 | 37.911 | < 0.001 | 1.830 | 2.030 | 3.574 | 4.4 |
|  | Shuman et al. 2019 | 2.990 | 0.0472 | 63.317 | < 0.001 | 2.897 | 3.083 | 3.579 | 4.4 |
|  | Shuman et al. 2020 (1) | 2.700 | 0.0672 | 40.193 | < 0.001 | 2.568 | 2.832 | 3.550 | 4.4 |
|  | Shuman et al. 2020 (2) | 2.990 | 0.0475 | 62.985 | < 0.001 | 2.897 | 3.083 | 3.579 | 4.4 |
|  | Shuman et al. 2023 | 2.900 | 0.0334 | 86.761 | < 0.001 | 2.834 | 2.966 | 3.593 | 4.4 |
|  | Söling et al. 2022 | 3.540 | 0.0654 | 54.144 | < 0.001 | 3.412 | 3.668 | 3.553 | 4.4 |
|  | Yıldız 2024 | 2.810 | 0.0470 | 59.818 | < 0.001 | 2.718 | 2.902 | 3.579 | 4.4 |

(Source: Authors own work)

Table S9. The effect sizes expressed as weighted mean differences and their 95% confidence intervals for the individual studies (ILS supportive).

|  | Study | Effect Size | Std. Error | Z | Sig. (2-tailed) | 95% Confidence Interval | | Weight | Weight (%) |
| --- | --- | --- | --- | --- | --- | --- | --- | --- | --- |
|  |  |  |  |  |  | Lower | Upper |  |  |
| Managers | Hsu et al.  2024 | 3.620 | 0.1266 | 28.602 | < 0.001 | 3.372 | 3.868 | 5.808 | 4.1 |
|  | Saiki et al. 2023 | 2.150 | 0.1148 | 18.722 | < 0.001 | 1.925 | 2.375 | 5.906 | 4.1 |
|  | Shuman et al. 2019 | 3.230 | 0.1126 | 28.686 | < 0.001 | 3.009 | 3.451 | 5.923 | 4.2 |
|  | Shuman et al. 2023 | 3.270 | 0.1087 | 30.074 | < 0.001 | 3.057 | 3.483 | 5.954 | 4.2 |
| Professionals | Aarons et al. 2014 | 2.630 | 0.0537 | 48.993 | < 0.001 | 2.525 | 2.735 | 6.288 | 4.4 |
|  | Aarons et al. 2016 | 2.900 | 0.0568 | 51.097 | < 0.001 | 2.789 | 3.011 | 6.275 | 4.4 |
|  | Boehm et al. 2022 | 3.700 | 0.0798 | 46.368 | < 0.001 | 3.544 | 3.856 | 6.153 | 4.3 |
|  | Borge et al. 2021 | 3.190 | 0.0600 | 53.147 | < 0.001 | 3.072 | 3.308 | 6.260 | 4.4 |
|  | Braathu et al. 2022 | 2.810 | 0.0340 | 82.531 | < 0.001 | 2.743 | 2.877 | 6.357 | 4.5 |
|  | Hsu et al.  2025 | 2.540 | 0.0474 | 53.642 | < 0.001 | 2.447 | 2.633 | 6.314 | 4.4 |
|  | Hu et al.  2021 | 2.870 | 0.0614 | 46.705 | < 0.001 | 2.750 | 2.990 | 6.253 | 4.4 |
|  | Kano et al. 2025 | 3.080 | 0.1355 | 22.725 | < 0.001 | 2.814 | 3.346 | 5.730 | 4.0 |
|  | J. Li et al.  2025 | 3.180 | 0.0250 | 127.302 | < 0.001 | 3.131 | 3.229 | 6.379 | 4.5 |
|  | L. Li et al. 2025 | 3.130 | 0.0165 | 189.805 | < 0.001 | 3.098 | 3.162 | 6.393 | 4.5 |
|  | Llarena et al. 2023 | 3.440 | 0.0567 | 60.706 | < 0.001 | 3.329 | 3.551 | 6.275 | 4.4 |
|  | Mandrou et al. 2020 | 3.160 | 0.0669 | 47.235 | < 0.001 | 3.029 | 3.291 | 6.226 | 4.4 |
|  | Saiki et al. 2023 | 2.000 | 0.0523 | 38.261 | < 0.001 | 1.898 | 2.102 | 6.294 | 4.4 |
|  | Shuman et al. 2019 | 3.030 | 0.0472 | 64.164 | < 0.001 | 2.937 | 3.123 | 6.314 | 4.4 |
|  | Shuman et al. 2020 (1) | 2.750 | 0.0651 | 42.273 | < 0.001 | 2.622 | 2.878 | 6.235 | 4.4 |
|  | Shuman et al. 2020 (2) | 3.030 | 0.0475 | 63.828 | < 0.001 | 2.937 | 3.123 | 6.313 | 4.4 |
|  | Shuman et al. 2023 | 2.970 | 0.0303 | 97.922 | < 0.001 | 2.911 | 3.029 | 6.367 | 4.5 |
|  | Söling et al. 2022 | 3.340 | 0.0867 | 38.523 | < 0.001 | 3.170 | 3.510 | 6.110 | 4.3 |
|  | Yıldız 2024 | 2.970 | 0.0432 | 68.756 | < 0.001 | 2.885 | 3.055 | 6.329 | 4.4 |

(Source: Authors own work)

Table S10. The effect sizes expressed as weighted mean differences and their 95% confidence intervals for the individual studies (ILS perseverant).

|  | Study | Effect Size | Std. Error | Z | Sig. (2-tailed) | 95% Confidence Interval | | Weight | Weight (%) |
| --- | --- | --- | --- | --- | --- | --- | --- | --- | --- |
|  |  |  |  |  |  | Lower | Upper |  |  |
| Managers | Hsu et al.  2024 | 2.890 | 0.1724 | 16.764 | < 0.001 | 2.552 | 3.228 | 4.294 | 3.9 |
|  | Saiki et al. 2023 | 1.580 | 0.1148 | 13.758 | < 0.001 | 1.355 | 1.805 | 4.622 | 4.2 |
|  | Shuman et al. 2019 | 2.880 | 0.1043 | 27.624 | < 0.001 | 2.676 | 3.084 | 4.672 | 4.2 |
|  | Shuman et al. 2023 | 2.930 | 0.0917 | 31.960 | < 0.001 | 2.750 | 3.110 | 4.727 | 4.3 |
| Professionals | Aarons et al. 2014 | 2.360 | 0.0583 | 40.449 | < 0.001 | 2.246 | 2.474 | 4.841 | 4.4 |
|  | Aarons et al. 2016 | 2.720 | 0.0590 | 46.117 | < 0.001 | 2.604 | 2.836 | 4.839 | 4.4 |
|  | Boehm et al. 2022 | 3.700 | 0.0798 | 46.368 | < 0.001 | 3.544 | 3.856 | 4.773 | 4.3 |
|  | Borge et al. 2021 | 2.820 | 0.0714 | 39.508 | < 0.001 | 2.680 | 2.960 | 4.802 | 4.4 |
|  | Braathu et al. 2022 | 2.490 | 0.0340 | 73.133 | < 0.001 | 2.423 | 2.557 | 4.894 | 4.4 |
|  | Hsu et al.  2025 | 2.380 | 0.0469 | 50.770 | < 0.001 | 2.288 | 2.472 | 4.870 | 4.4 |
|  | Hu et al.  2021 | 2.520 | 0.0667 | 37.793 | < 0.001 | 2.389 | 2.651 | 4.817 | 4.4 |
|  | Kano et al. 2025 | 3.110 | 0.1345 | 23.124 | < 0.001 | 2.846 | 3.374 | 4.520 | 4.1 |
|  | J. Li et al.  2025 | 3.160 | 0.0259 | 122.088 | < 0.001 | 3.109 | 3.211 | 4.906 | 4.5 |
|  | L. Li et al. 2025 | 3.000 | 0.0169 | 177.257 | < 0.001 | 2.967 | 3.033 | 4.915 | 4.5 |
|  | Llarena et al. 2023 | 3.300 | 0.0592 | 55.775 | < 0.001 | 3.184 | 3.416 | 4.839 | 4.4 |
|  | Mandrou et al. 2020 | 3.180 | 0.0552 | 57.617 | < 0.001 | 3.072 | 3.288 | 4.846 | 4.4 |
|  | Saiki et al. 2023 | 1.790 | 0.0514 | 34.850 | < 0.001 | 1.689 | 1.891 | 4.859 | 4.4 |
|  | Shuman et al. 2019 | 2.840 | 0.0496 | 57.277 | < 0.001 | 2.743 | 2.937 | 4.863 | 4.4 |
|  | Shuman et al. 2020 (1) | 2.680 | 0.0686 | 39.073 | < 0.001 | 2.546 | 2.814 | 4.811 | 4.4 |
|  | Shuman et al. 2020 (2) | 2.840 | 0.0498 | 56.977 | < 0.001 | 2.742 | 2.938 | 4.863 | 4.4 |
|  | Shuman et al. 2023 | 2.740 | 0.0347 | 79.047 | < 0.001 | 2.672 | 2.808 | 4.893 | 4.4 |
|  | Söling et al. 2022 | 3.080 | 0.0689 | 44.680 | < 0.001 | 2.945 | 3.215 | 4.810 | 4.4 |
|  | Yıldız 2024 | 2.690 | 0.0400 | 67.324 | < 0.001 | 2.612 | 2.768 | 4.884 | 4.4 |

(Source: Authors own work)
